# Correction of Gingival Architecture Using the Biologically Oriented Preparation Technique in Two Patients with Human Immunodeficiency Virus

**DOI:** 10.1155/2020/8830949

**Published:** 2020-12-14

**Authors:** Luca Casula, Alex Gillone, Davide Musu

**Affiliations:** ^1^Vita Salute University, Milan, Italy; ^2^East Carolina University, Greenville, USA; ^3^Department of Endodontology, Academic Centre for Dentistry Amsterdam (ACTA), Amsterdam, Netherlands

## Abstract

Two female patients positive for human immunodeficiency virus and receiving antiretroviral treatment presented with esthetic concerns due to fixed dental prostheses in the anterior region. The patients had gingival recession and short clinical crowns in the esthetic zone. In both cases, the biologically oriented preparation technique was used to recreate ideal proportions between the teeth and gingivae. Since patients with human immunodeficiency virus experience a progressive deterioration of their immune systems and other significant comorbidities, a reduction in the number of traumatic clinical procedures is recommended. The use of this minimally invasive prosthetic protocol has not been previously reported in patients with human immunodeficiency virus. These cases demonstrate how short clinical crowns and gingival recession in the esthetic zone can be successfully treated with the biologically oriented preparation technique to avoid surgical procedures in patients with human immunodeficiency virus.

## 1. Introduction

Various studies have shown that individuals with human immunodeficiency virus (HIV) have a similar risk of complications after invasive dental procedures as other patients [1, 2]. Moreover, when present, these complications tend to be minor and are easily treated [3]. This finding has led to an increased demand among this patient population for oral health care, including periodontal surgery for the treatment of gingival recession [4]. Gingival recession can occur in crown-restored teeth and may induce root hypersensitivity, root caries, and esthetic concerns, especially if present in the anterior region [5, 6]. A short clinical crown associated with an excessive amount of maxillary gingival display can contribute to a negative self-image in patients and may even have negative social ramifications [7]. Both short clinical crowns and gingival recession are generally treated with periodontal surgical procedures. Short clinical crowns are treated with crown lengthening, which is often a part of a multidisciplinary approach [7, 8], while root coverage procedures are used to manage gingival recession [9].

The biologically oriented preparation technique (BOPT) is a prosthetic procedure useful for the retreatment of fixed prostheses with gingival recession [10–12]. This procedure involves a feather-edge subgingival preparation [13], in which the prosthetic crown margin is positioned according to the desired location of the gingival margin and serves as the new cementoenamel junction [10]. The BOPT protocol allows for the thickening of the soft tissues in the coronal direction and results in excellent esthetics with both implants [14, 15] and natural teeth [11, 12].

There are no clinical case reports documenting the use of the BOPT in patients with HIV. In this report, we present two cases in which the BOPT was successfully used to treat both gingival recessions and short clinical crowns in HIV-positive patients with fixed dental prostheses. The BOPT was used to recreate gingival symmetry and harmony, thus avoiding periodontal surgical procedures.

## 2. Case Presentation

### 2.1. Case Report 1

A 55-year-old female patient with HIV (cluster of differentiation (CD4) count of 410 cells/*μ*L and an undetectable viral load) was undergoing antiretroviral treatment (Triumeq, Tivicay, Lamivudina) at our institution. She was referred to the Dentistry Department of the same hospital in order to address her oral esthetic concerns. The patient was unsatisfied with the appearance of her smile due to a previous interim full-arch rehabilitation (right first to left first maxillary molars) and multiple areas of gingival recession (Figure 1(a)).

The proposed treatment plan included the replacement of the old interim fixed restoration with a new full-arch restoration, with the use of the BOPT to recreate proper teeth proportions and gingival symmetry and harmony [10–13]. The gingival height and thickness were measured prior to the prosthetic procedure. The gingival height was measured on the buccal side with a dental probe from the mucogingival junction to the gingival sulcus in the center of each tooth. The gingival thickness was measured in each tooth by inserting a K-file with an endodontic stop 1 mm below the gingival margin in the middle of the buccal side of the keratinized gingiva. The existing prosthesis was then removed, and the sulci were probed to establish the depth of the subgingival preparation [10]. A flame-shaped bur was then inserted into the gingival sulcus, and the preexisting finish lines were eliminated from the abutment teeth [13].

The patient had a previous medical history of temporomandibular joint (TMJ) disorder, which had resolved after provisional prosthetic treatment. The decision was made to maintain the same interim fixed complete arch restoration during the provisional stage to avoid changes in the occlusal relationship and to reduce costs. Thus, the interim full-arch restoration was relined with acrylic resin (Sintodent, Sintodent s.r.l. Italia, Rome, Italy), and the crown margins were positioned 0.5 subgingivally [10–13]. After 1 month of soft tissue maturation, the maxillary left lateral incisor was shortened to leave the gingival recession uncovered (Figure 2); this was because gingival adaptation to the new prosthetic emergence profile was expected, as described by previous studies utilizing the BOPT [10]. A collagen matrix graft (equine collagen-BioPad) was placed with a minimal partial thickness flap in the edentulous buccal area of the pontic zones (Figure 1(b)).

One month later, the gingival recession of the left lateral incisor was reduced, and a month after that, the gingiva was completely adapted to the new crown shape (Figure 2). The gingival margin of the left lateral incisor, which was apical to that of the adjacent teeth before the BOPT (Figure 3(a)), was now in a more coronal position (Figure 3(b)); the gingiva appeared thicker than before, thus achieving a correct gingival architecture with a healthy gingival sulcus (Figure 3(c)). After 3 months of soft tissue maturation, the definitive impressions (Putty and Light Elite HD, Zhermack, Italy) were taken and sent to the laboratory for fabrication of a metal framework for the definitive full-arch metal-ceramic restoration (from maxillary right first to left first molars). At the next appointment, the fit of the metal framework was assessed using a dual-cured paste (Fit Checker Advanced, GC, Tokyo, Japan). Also, the framework-soft tissue relationship was captured with a framework transfer impression that was sent to the laboratory for veneering, and the final restoration was cemented.

At the 24-month follow-up, the supporting tissues around the fixed prosthesis were healthy, with no signs of periodontal disease (Figure 4(a)). The gingiva appeared pink and stippled, and complete papillae fill was obtained at the interdental spaces (Figures 4(b) and 4(c)). The height and the thickness of the keratinized gingiva were also remeasured at the 24-month follow-up. The gingival height was found to be stable for all teeth, except for the left lateral incisor, which was augmented from 2 mm before teeth preparation to 3.5 mm at the 24-month follow-up. The gingival thickness increased from a mean value of 1.16 mm to 1.69 mm.

Adequate oral health care and the maintenance of a pleasant smile with esthetic restorations may improve the quality of life in HIV-positive patients [16]. However, due to progressive immunocompromise, it may be preferable to reduce the number of traumatic clinical procedures in patients diagnosed with HIV.

### 2.2. Case Report 2

A 45-year-old female patient undergoing treatment (Triumeq, Tivicay, Lamivudina) for HIV infection (CD4 count of 1313 cells/*μ*L and an undetectable viral load) at our institution was referred to the Dentistry Department for her oral esthetic concerns. Clinical examination revealed short clinical crowns on the maxillary incisors, which the patient reported to be the cause of her negative self-image (Figure 5(a)).

The proposed treatment included the removal of the old anterior crown restorations (maxillary central incisors and left lateral incisors) and the placement of four zirconia ceramic complete crowns (left lateral incisor to left lateral incisor) using the BOPT [15]. Treatment goals included the correction of the dimensions and proportions of the maxillary incisors and the reestablishment of the correct gingival architecture. Two alginate impressions and a centric relation occlusal record were taken and sent to the dental laboratory for the fabrication of a resin interim fixed dental prosthesis (from maxillary right to left lateral incisors). Before starting the prosthetic procedure, the gingival height and thickness were measured as in the previous case. The teeth were then prepared with a flamed-shaped bur inserted to the bottom of the sulcus [10]. The interim restoration was relined and placed no deeper than 0.5 to 1 mm subgingivally [10–13].

One month later, the gingival tissue had healed (Figure 5(b)), and the gingival sulcus of the central incisors was remeasured. It was found to be 2 mm in the midbuccal and midpalatal sites and 3 mm in the interproximal sites of the central incisors. As a result, the apico-coronal dimension of the interim restoration was increased by approximately 1.5 mm, only on the buccal aspect. The restoration extended subgingivally and 0.5 mm coronally from the bottom of the previously measured buccal gingival sulcus, such that the prepared gingiva was pushed on the buccal side and displaced apically to the same extent. One month after soft tissue maturation, the gingiva appeared healthy at both teeth, and the gingival margins of the central incisors were at the same level as those of the canines (Figure 6(a)). The definitive impression was taken (Putty and Light Elite HD, Zhermack, Italy), and a zirconia framework was fabricated by the dental laboratory and veneered after a clinical check. The buccal prosthetic emergence profiles of the definitive restorations were slightly overcontoured in order to support the matured soft tissues and were fabricated based on the conditioned soft tissues during the interim stage with the buccal portion 1.5 mm more apical than the palatal portion.

At the 12-month follow-up, the keratinized gingiva appeared pink and stippled, and the interdental spaces were fully filled by interdental papillae (Figure 6(b)). No abnormal periodontal findings, such as bleeding on probing, increased probing depth, or inflammation, were detected. The height and the thickness of the keratinized gingiva were also remeasured at the 12-month follow-up. The gingival height was found to be stable at the maxillary lateral incisors and decreased from 4 mm at the central incisors before teeth preparation to 2 mm at the 12-month follow-up. The gingival thickness increased from a mean value of 1.44 mm to 1.87 mm.

## 3. Discussion

Thus, in the present case reports, gingival recession and short clinical crowns were treated only by means of a prosthetic approach called the BOPT. Feather-edge preparations have previously been suspected to cause damage to the periodontal tissue because of the overcontoured profile of the crown [17]; however, this technique also offers a good marginal fit that is of fundamental importance for gingival health [18–20]. Moreover, the use of the preparation without a finish line in the BOPT protocol seems to increase gingival thickness, achieving stable and healthy periodontal tissues [10–13]. The feather-edge preparation creates wide interdental spaces with wide embrasures in both provisional and definitive restorations [21]. These characteristics are important because they allow the patients to easily clean the interdental spaces. However, during the interim stage and sometimes even the final stage, the restorations may present empty interdental spaces that can make the smile unattractive if present in the esthetic zone. Nevertheless, in these cases, the interdental spaces were completely closed with elongated interdental papillae, as in the natural dentition (Figures 4 and 6). Moreover, an increase in the gingival tissue thickness was detected at the 24-month follow-up in the first case and at the 12-month follow-up in the second case. In the first case, the gingival height, measured in relation to the gingival recession of the lateral incisor, increased at the 24-month follow-up. However, in the second case, the gingival height decreased at the 12-month follow-up.

This report describes the use of the BOPT protocol in patients with HIV for retreatment of fixed dental prostheses causing esthetic concerns. The technique was used successfully to treat gingival recession and short clinical crowns, by recreating the ideal proportions between the teeth and gingivae. The use of the BOPT may allow dentists to avoid surgical procedures in these patients, who are experiencing a progressive decline in their immune function and other significant comorbidities.

## Figures and Tables

**Figure 1:**
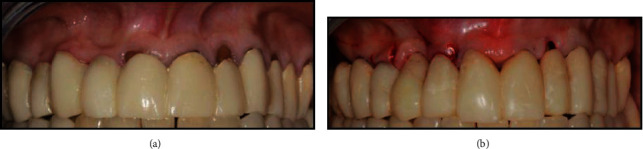
(a) Preoperative intraoral view. (b) The interim full-arch restoration after shortening of the left lateral incisor and the collagen matrix placement.

**Figure 2:**
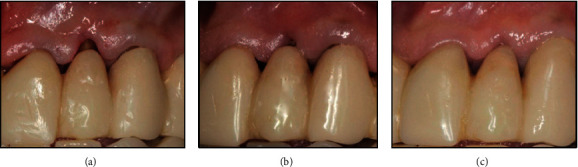
Adaptation of the gingiva to the new prosthetic emergence profile (a) interim restoration on the shortened left lateral incisor, (b) reduction of the gingival recession after 1 month, and (c) gingiva completely adapted to the left lateral incisor after 2 months.

**Figure 3:**
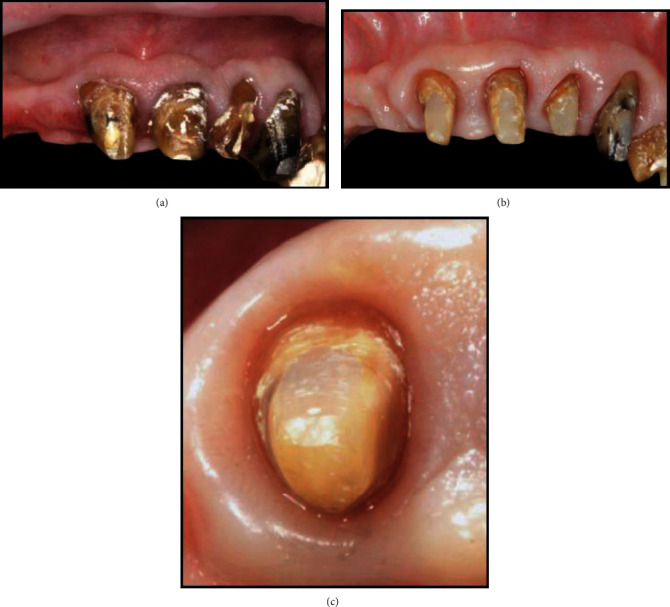
(a) Abutment teeth and gingival architecture before the biologically oriented preparation technique (BOPT) protocol. (b) Abutment teeth and gingival architecture after the BOPT protocol. (c) Widening of the abutment tooth and gingival sulcus.

**Figure 4:**
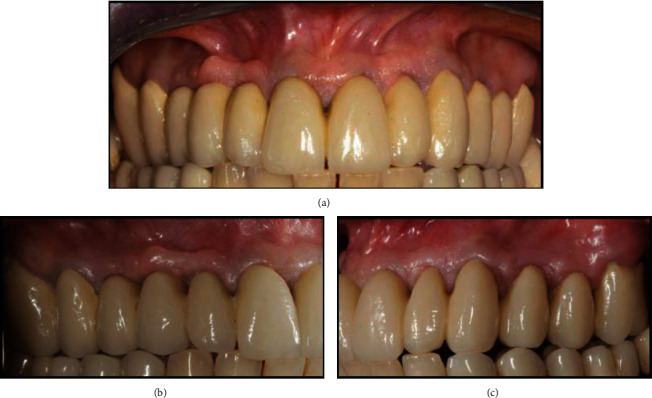
(a) Final rehabilitation at the 24-month follow-up: facial view. (b) Final rehabilitation at the 24-month follow-up: right lateral view. (c) Final rehabilitation at the 24-month follow-up: left lateral view.

**Figure 5:**
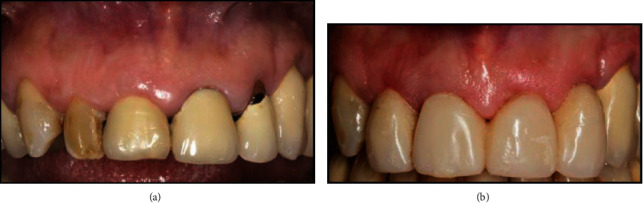
(a) Preoperative intraoral view. (b) Interim restoration after 1 month of healing after the biologically oriented preparation technique.

**Figure 6:**
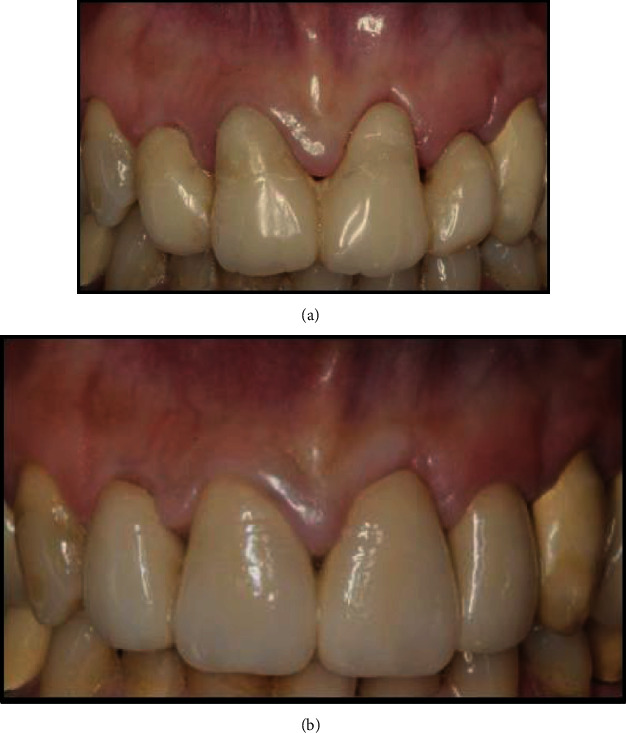
(a) The displacement of the buccal gingiva after vertical augmentation of the emergence profile of the central incisors. (b) Definitive restoration at 12-month follow-up.

## Data Availability

The data that support the findings of this study are available from the corresponding author upon reasonable request.
